# Enhanced Biocatalytic Esterification with Lipase-Immobilized Chitosan/Graphene Oxide Beads

**DOI:** 10.1371/journal.pone.0104695

**Published:** 2014-08-15

**Authors:** Siaw Cheng Lau, Hong Ngee Lim, Mahiran Basri, Hamid Reza Fard Masoumi, Asilah Ahmad Tajudin, Nay Ming Huang, Alagarsamy Pandikumar, Chi Hua Chia, Yoshito Andou

**Affiliations:** 1 Department of Chemistry, Faculty of Science, Universiti Putra Malaysia, Serdang, Selangor, Malaysia; 2 Institute of Advanced Technology, University Putra Malaysia, Serdang, Selangor, Malaysia; 3 Laboratory of Molecular Biomedicine, Institute of Bioscience, Universiti Putra Malaysia, Serdang, Selangor, Malaysia; 4 Faculty of Biotechnology and Biomolecular Sciences, Universiti Putra Malaysia, Serdang, Selangor, Malaysia; 5 Low Dimensional Materials Research Centre, Department of Physics, Faculty of Science, University of Malaya, Kuala Lumpur, Malaysia; 6 School of Applied Physics, Faculty of Science and Technology, Universiti Kebangsaan Malaysia, Bangi, Selangor, Malaysia; 7 Eco-Town Collaborative Research and Development Center for the Environment and Recycling, Kyushu Institute of Technology, Kitakyushu-shi, Fukuoka, Japan; Russian Academy of Sciences, Institute for Biological Instrumentation, Russian Federation

## Abstract

In this work, lipase from *Candida rugosa* was immobilized onto chitosan/graphene oxide beads. This was to provide an enzyme-immobilizing carrier with excellent enzyme immobilization activity for an enzyme group requiring hydrophilicity on the immobilizing carrier. In addition, this work involved a process for the preparation of an enzymatically active product insoluble in a reaction medium consisting of lauric acid and oleyl alcohol as reactants and hexane as a solvent. This product enabled the stability of the enzyme under the working conditions and allowed the enzyme to be readily isolated from the support. In particular, this meant that an enzymatic reaction could be stopped by the simple mechanical separation of the “insoluble” enzyme from the reaction medium. Chitosan was incorporated with graphene oxide because the latter was able to enhance the physical strength of the chitosan beads by its superior mechanical integrity and low thermal conductivity. The X-ray diffraction pattern showed that the graphene oxide was successfully embedded within the structure of the chitosan. Further, the lipase incorporation on the beads was confirmed by a thermo-gravimetric analysis. The lipase immobilization on the beads involved the functionalization with coupling agents, N-hydroxysulfosuccinimide sodium (NHS) and 1-ethyl-(3-dimethylaminopropyl) carbodiimide (EDC), and it possessed a high enzyme activity of 64 U. The overall esterification conversion of the prepared product was 78% at 60°C, and it attained conversions of 98% and 88% with commercially available lipozyme and novozyme, respectively, under similar experimental conditions.

## Introduction

Esterification is widely used for the synthesis of raw materials as emulsifiers in foods, lubricants, paints, emollients in cosmetics, and in perfumes [1]. Lipase is one of the biocatalysts that catalyzes an esterification process such as the hydrolysis of fats or lipids [2]. Lipase-catalyzed esterification is a vital reaction because of its useful ester products. More specifically, lipase-catalyzed esterification reactions have attracted research interest due to an increased use of organic esters in biotechnology and the chemical industry [3]. Lipase consists of two different structures in a homogeneous solution. One is a flap structure with its active site separated from the reaction medium, and the other is an open structure with its active site exposed to the reaction medium [4], [5]. Homogeneous biocatalysis has some drawbacks related to the recovery of the catalyst and disposal of the wastewater. However, these problems can be overcome by using a heterogeneous solid catalyst because it will be more stable against different pH and temperature ranges [5]–[8]. It can also be continuously reused in various reactors [9]. Moreover, lipase immobilization is used to prevent product contamination, ease recovery, enhance the operational lifetime and stability, prevent the solvent denaturation of the lipase, and expose the active sites of the lipase for more efficient bonding with a support [6], [10]–[12].

Generally, two methods are commonly employed for the enzyme immobilization: chemical methods involving covalent bonds with the enzyme and physical methods involving weak interactions with the enzyme [13], [14]. Immobilization using covalent binding is more favorable because it provides a strong bonding between the lipase and support. In addition, covalent binding eases the reuse of the lipase more efficiently than physical methods like adsorption and entrapment [10]. Various supports have been used for the immobilization of lipase [15]. These encompass inorganic materials such as diatomaceous earth, silica and porous glass, synthetic resins and resin ion exchangers, and natural polysaccharides such as cellulose and cross-linked dextrin [15]. Chitosan is a natural polymer obtained by the deacetylation of chitin in a strongly basic medium at elevated temperature. It is insoluble in water [16] and can be easily reformed and reshaped from its solution by the addition of an alkali [17]. Chitosan is a more suitable support to immobilize the enzyme because it has a low cost and is biocompatible, nontoxic, available in various forms, physiological inert, and has high mechanical strength [11], [18]–[20].

Immobilizing lipase on chitosan enhances the enzyme stability and eases the removal of the enzyme from the support without inactivating the enzyme or contaminating the support [16]. Chitosan beads can be prepared as a support for the immobilization of an enzyme by precipitating an acidic chitosan solution into a sodium hydroxide solution and then performing a solvent exchange with water [17]. Larger pores are more uniformly present from the surface to the inside of the carrier when chitosan is used as a carrier rather than synthetic resin carriers, thus providing greater support diffusibility [15]. Chitosan is a useful polysaccharide in protein conjugation via cross-linking agents such as N-(3-dimethylaminopropyl)-N-ethylcarbodiimide (EDC) [21]. One of the uses of EDC is to activate the hydroxyl groups of chitosan for the immobilization of lipase [22]. The highly reactive amino groups convenient for immobilizing enzymes via covalent bonding are disposed within the molecule [15]. In addition, chitosan has a higher affinity to enzymes because a higher amount of the enzymes may be immobilized onto the highly reactive amino groups of chitosan, compared to inorganic materials and synthetic resins [15].

An aerogel of chitosan beads embedded with graphene oxide (GO) obtained from freeze-drying is a type of matter that has an open cell foam structure with ultrafine pores and a cell size that results in a large surface area [23]. GO is able to enhance the physical strength of chitosan beads because of its superior mechanical integrity and low thermal conductivity, which are important properties for a catalyst support [23]. Moreover, the oxide functional groups of GO can provide additional active sites for the immobilization of lipase. Lipase is covalently immobilized onto chitosan beads embedded with GO (CS/GO) before they are used for esterification. Upon immobilization, the coiled structure of lipase will be expanded, hence exposing its active sites and promoting effective bonding with the support.

In the present study, the lipase from *Candida rugosa* was immobilized on chitosan/graphene oxide beads that were prepared and characterized using suitable techniques, which are field emission scanning electron microscopy, an X-ray diffraction technique, a thermogravimetric analysis, and a lipase assay [29]. The lipase immobilization on the beads involved functionalization with coupling agents, N-hydroxysulfosuccinimide sodium (NHS) and 1-ethyl-(3-dimethylaminopropyl) carbodiimide (EDC), and it possessed a high enzyme activity of 64 U. The commercially available lipases that were used for the reaction was a lipase of *Rhizomucor miehei* immobilized on a macroporous anion exchange resin (Lipozyme RM IM) and a lipase of *Candida antartica* Lipase B immobilized on acrylic resin (Novozyme 435). They are both currently being used in biodiesel productions [24], [25]. The overall esterification conversion of the prepared product was 78% at 60°C, and it reached 98% and 88% with commercially available lipozyme and novozyme, respectively, under similar experimental conditions.

## Experimental Methods

### 1.1 Materials and characterization techniques

High purity sodium hydroxide (NaOH), ammonium hydroxide (NH_4_OH, 25%), and acetic acid were purchased from Merck. Chitosan with a 75%–85% degree of deacetylation was purchased from Sigma Aldrich. Graphite flakes were purchased from Ashbury Graphite Mill Inc. The characterizations of the biocatalysts were performed using an FEI Nova NanoSEM 400 field emission scanning electron microscope (FESEM), Philips X-Ray diffractometer (XRD), Renishaw inVia Raman microscope, and TA-Q500 thermo-gravimetric analyzer.

### 1.2 Synthesis of chitosan/graphene oxide beads

The chitosan/graphene oxide beads were prepared using the following procedure. Initially, a chitosan solution (2% (w/v) of chitosan in a 2.5% acetic acid solution) was added drop-wise to a coagulant bath containing a 1 M NaOH solution, and then the beads were obtained after 4 h of coagulation. The chitosan beads were washed 5 times with deionized water in order to remove the excess acetic acid and NaOH. Then, the chitosan beads (100 µg/mL) were mixed with GO. The GO was prepared using a simplified Hummer's method [26] and was reduced in size by sonication using a 600 W horn sonicator for 1 h (represented as CS/GO-S beads). For comparison, the same protocol was used to prepare CS/GO beads without sonication. The beads were washed and freeze-dried for 24 h.

### 1.3 Functionalization of chitosan/graphene oxide beads

A 50 mg quantity of the chitosan/graphene oxide beads was dispersed into 50 mL of phosphate buffer saline (PBS) solution (pH 7), and then N-Hydroxysulfosuccinimide sodium (NHS) was added. The mixture was subjected to sonication for 15 min, and then 1-ethyl-(3-dimethylaminopropyl) carbodiimide (EDC) (20 mmol/L) was added. The resulting mixture was shaken at 200 rpm for 60 min. The activated solution was then filtered through filter paper and rinsed thoroughly with PBS buffer to remove the excess EDC and NHS.

### 1.4 Immobilization of lipase with functionalized beads

The functionalized chitosan/graphene oxide beads were transferred to a solution of lipase (5 mg/mL). The immobilization of the lipase was performed at 25°C with 150 rpm stirring for 15 h. After conjugation, the mixture was filtered using filter paper and 6 washes were performed typically, with PBS solution added each time to remove the unbound lipase. The lipase immobilized with CS/GO with the aid of coupling agents is denoted as CS/GO-CL, and the lipase immobilized with CS/GO-S with the aid of coupling agents is denoted as CS/GO-SCL. As a positive control, CS/GO not treated with a coupling agent was prepared and used to immobilize lipase, and is denoted as CS/GO-L. Similarly, CS/GO-S not treated with a coupling agent was prepared and used to immobilize lipase, and is denoted as CS/GO-SL. Likewise, CS is denoted as CS-L and CS-CL upon the immobilization of lipase in the absence and presence of coupling agents.

### 1.5 Esterification of lauric acid and oleyl alcohol

#### 1.5.1 Water bath method

The reaction mixture consisted of lauric acid (1 mmol), oleyl alcohol (1 mmol), and a biocatalyst (0.5 w/w). Hexane was added to make a total volume of 10 mL. The reaction mixture was incubated in a horizontal shaker water bath at a speed of 150 rpm at 40°C for 24 h [27]. All of the experiments were performed in triplicate, and control experiments were performed without biocatalysts.

#### 1.5.2 Oil bath method

The reaction mixture consisted of lauric acid (1 mmol), oleyl alcohol (1 mmol), and a biocatalyst (0.5 w/w). Hexane was added to make a total volume of 10 mL. The reaction mixture was stirred in an oil bath at 60°C for 4 h [28]. All of the experiments were conducted in triplicate, and control experiments were performed without biocatalysts.

### 1.6 Analysis

The reaction was terminated by dilution with 3 mL of ethanol:acetone (50∶50, v/v), and the biocatalyst was filtered through filter paper. The unreacted lautic acid in the reaction mixture was determined by titration with 0.1 M NaOH using a pH meter to an end point of pH 9.9±0.1. The activity of the biocatalyst was expressed as a conversion percentage, as shown in Eq. (1).
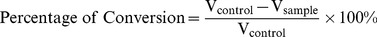
(Eq. 1)


Where 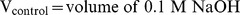 needed to titrate the control and 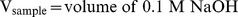 needed to titrate the sample.

The biocatalyst activity was determined by using the Kwon and Rhee method [29]. First, a substrate was prepared using olive oil and phosphate buffer (1∶1 ratio, pH 7, 50 mM). A control was prepared by mixing 0.02 mL of CaCl_2_, 2.5 mL of the olive oil–PBS mixture, and 1 mL of the PBS solution. A sample contained 0.02 mL of CaCl_2_, 2.5 mL of the olive oil–PBS mixture, 1 mL of the PBS solution, and 5 mg of the biocatalyst. The mixture was then incubated at 20°C and 200 rpm for 30 min in a horizontal shaker water bath. Then, 1 mL of HCl and 5 mL of isooctane were added to each of the reaction mixtures and vortexed for 30 s. After the mixture had settled down and two layers formed, 4 mL of the upper layer was transferred into a new test tube, and 1 mL of copper pyridine was added. It was then vortexed for 30 s, and the mixture was allowed to settle for 30 min. A 1 mL quantity of the mixture was then placed into a cuvette, and absorbance reading at 715 nm was recorded in triplicate. A blank test was carried out using isooctane. The required formula for calculating the enzyme activity is shown in Eq. (2).
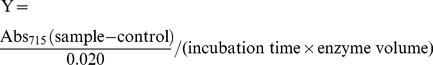
(Eq. 2)


## Results and Discussion

**Figure 1:**
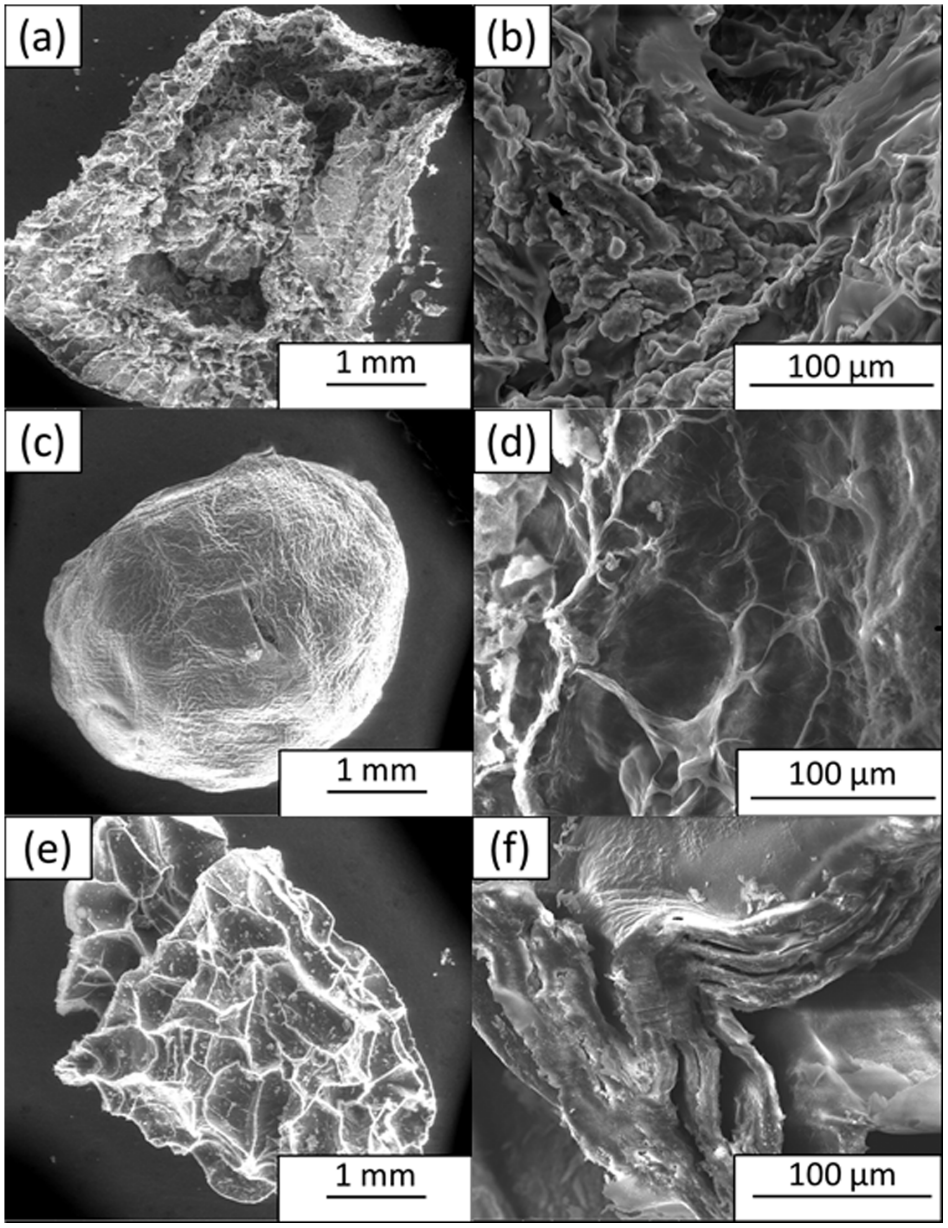
FESEM images of CS beads after coagulation time intervals of (a and b) 2 h, (c and d) 4 h, and (e and f) 20 h.

The morphologies of the chitosan beads with coagulation periods of 2, 4, and 20 h were studied using the FESEM and are shown in **Fig. 1**. The beads prepared after 2 h of coagulation were fragile and lacked a definite structure (**Fig. 1a**). At a microscopic level, in which 2000× magnification was used, the beads were found to lack porosity and had a featureless appearance (**Fig. 1b**). When the coagulation time was doubled, the beads reflected a much improved structure, which was firm and spherical, indicating an optimum aging time for the formation of well-defined spherical beads (**Fig. 1c**). A higher degree of porosity at the micron scale resulting from scaffolds made up of paper-like thin walls of concave pockets could be observed (**Fig. 1d**). At a prolonged coagulation time of 20 h, the beads shrunk and lost their spherical integrity (**Fig. 1e**). Upon scrutiny, the morphology of the beads appeared to be in layers, suggesting that they once had thin walls with a continuous interconnected structure that had collapsed, causing the pores to be indistinguishable (**Fig. 1f**).

**Figure 2:**
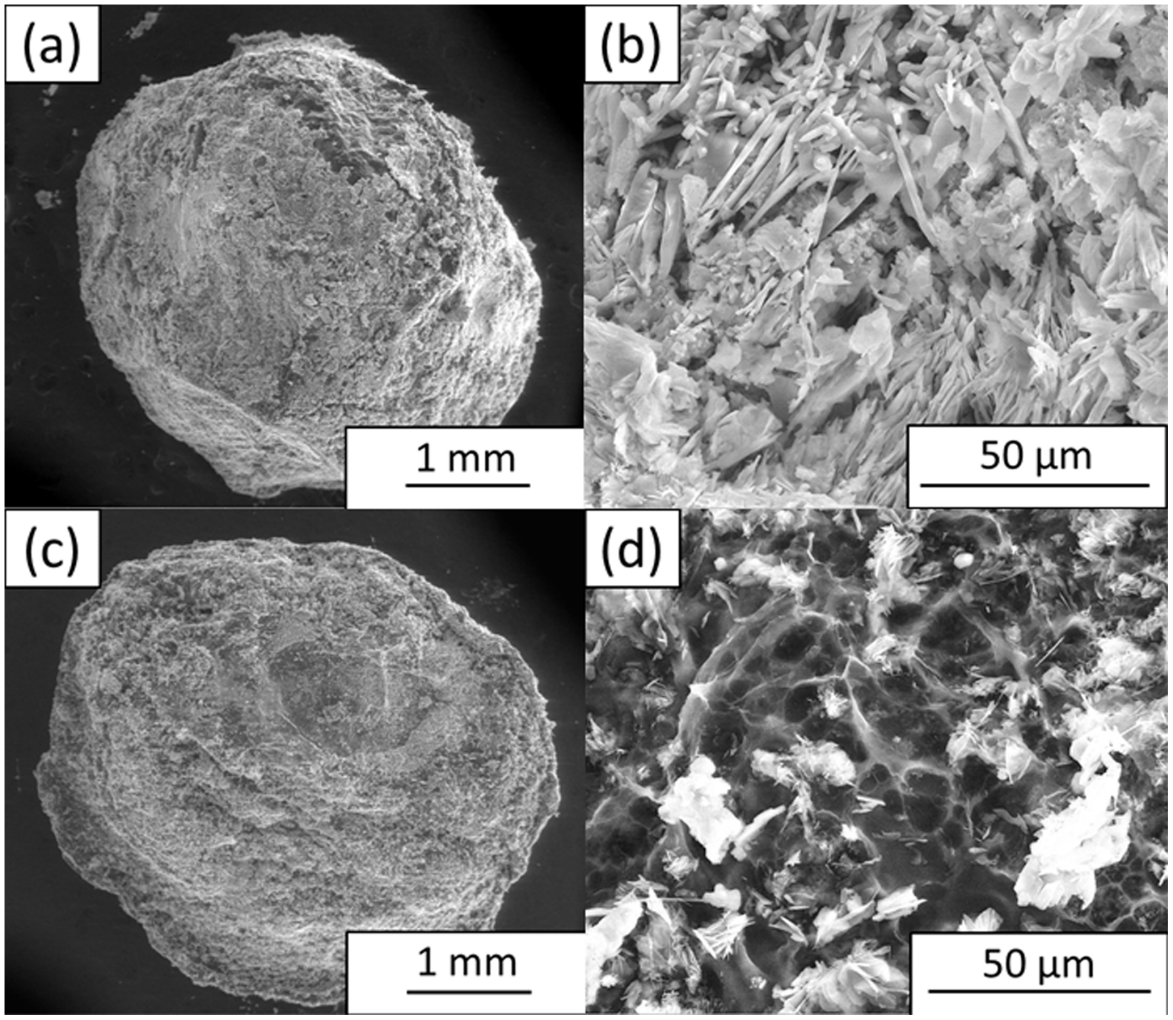
FESEM images of (a and b) CS/GO-S and (c and d) CS/GO beads coagulated for 4 h.

**Figure 3:**
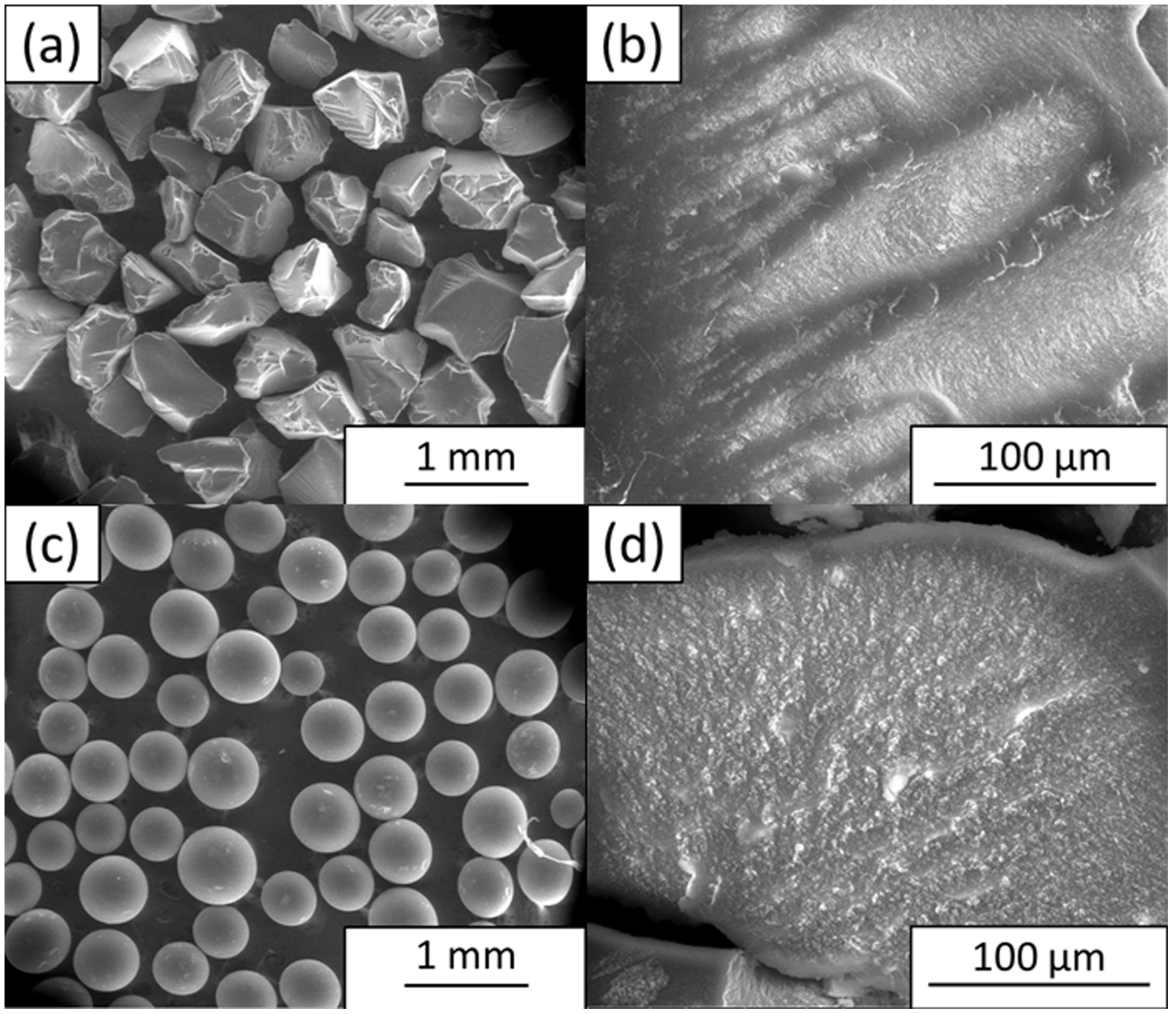
FESEM images of commercial (a and b) lipozyme and (c and d) novozyme.

Even though the CS beads prepared at the optimized coagulation time of 4 h showed better formed spherical beads, they failed to maintain their mechanical strength because they were brittle when touched. Therefore, the CS beads were incorporated with GO to enhance their mechanical structure. The inclusion of GO changed the morphology of the beads, as shown in **Fig. 2**. The CS/GO-S bead had a more rigid and spherical structure (**Fig. 2a**), and its FESEM image (**Fig. 2b**) shows a spiky structure that resulted from the inclusion of sonicated GO nanoparticles, which could further enhance its mechanical strength. The CS/GO bead had a less rigid spherical structure (**Fig. 2c**), and its FESEM image (**Fig. 2d**) shows concave pockets with smaller pores compared to those of **Fig. 1d**. It also shows some nanostructures, which resulted from the presence of a small portion of GO nanosheets. For comparison purposes, the FESEM images for commercially available lipozyme and novozyme were recorded and are shown in **Fig. 3**. It can be seen from **Fig. 3(a and b)** that the lipozyme has an irregular polygonal shape, and it has an uneven surface with a compact structure, which is very different from the highly porous structure of the prepared beads. Novozyme has a definite round shape (**Fig. 3(c and d)**) with a relatively smooth surface and is as compact as the lipozyme. These two commercial biocatalysts are dense and completely absent of pores, which are in contrast with the prepared biocatalysts.

**Figure 4:**
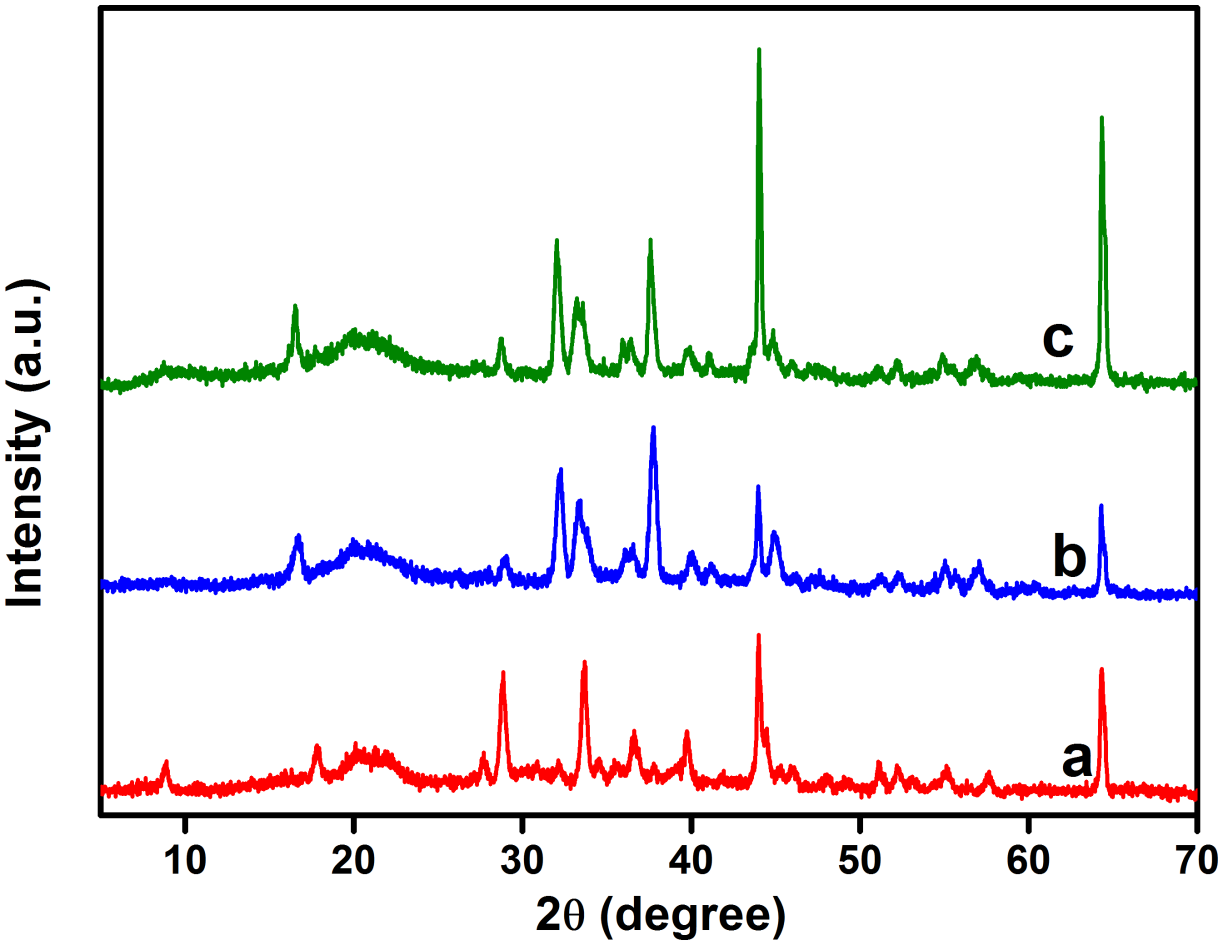
XRD patterns obtained for (a) CS, (b) CS/GO, and (c) CS/GO-S beads.

In **Fig. 4**, the XRD profile of CS presents two intense peaks at 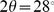 and 34°, which are assigned to JCPDS card No: 039–1894. The XRD profiles of both CS/GO and CS/GO-S differ only slightly from that of CS, which indicates that the GO nanosheets were exfoliated and uniformly dispersed within the polymer matrix. The typical peak of GO at around 9°–11° is missing [30], evidencing the complete mixing of GO within the matrix of CS. The XRD results indicate that a significant amount of the amino groups of CS were inserted between the GO layers. After an amino nucleophilic substitution reaction with the epoxy groups of GO, the exfoliation of GO occurred. Hence, the destruction of its layered structure resulted in the disappearance of the typical peak of GO [31]. Furthermore, the incorporation of the fillers did little to affect the crystalline structure of CS because of the low GO content. However, the peak at 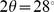 was greatly reduced in intensity for the nanocomposites, implying that the crytalline plane was altered by the presence of GO. Moreover, the sharp peak at 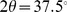 indicated that the regular stack of GO was broken, in addition to the absence of a graphite peak at 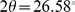 [32].

**Figure 5:**
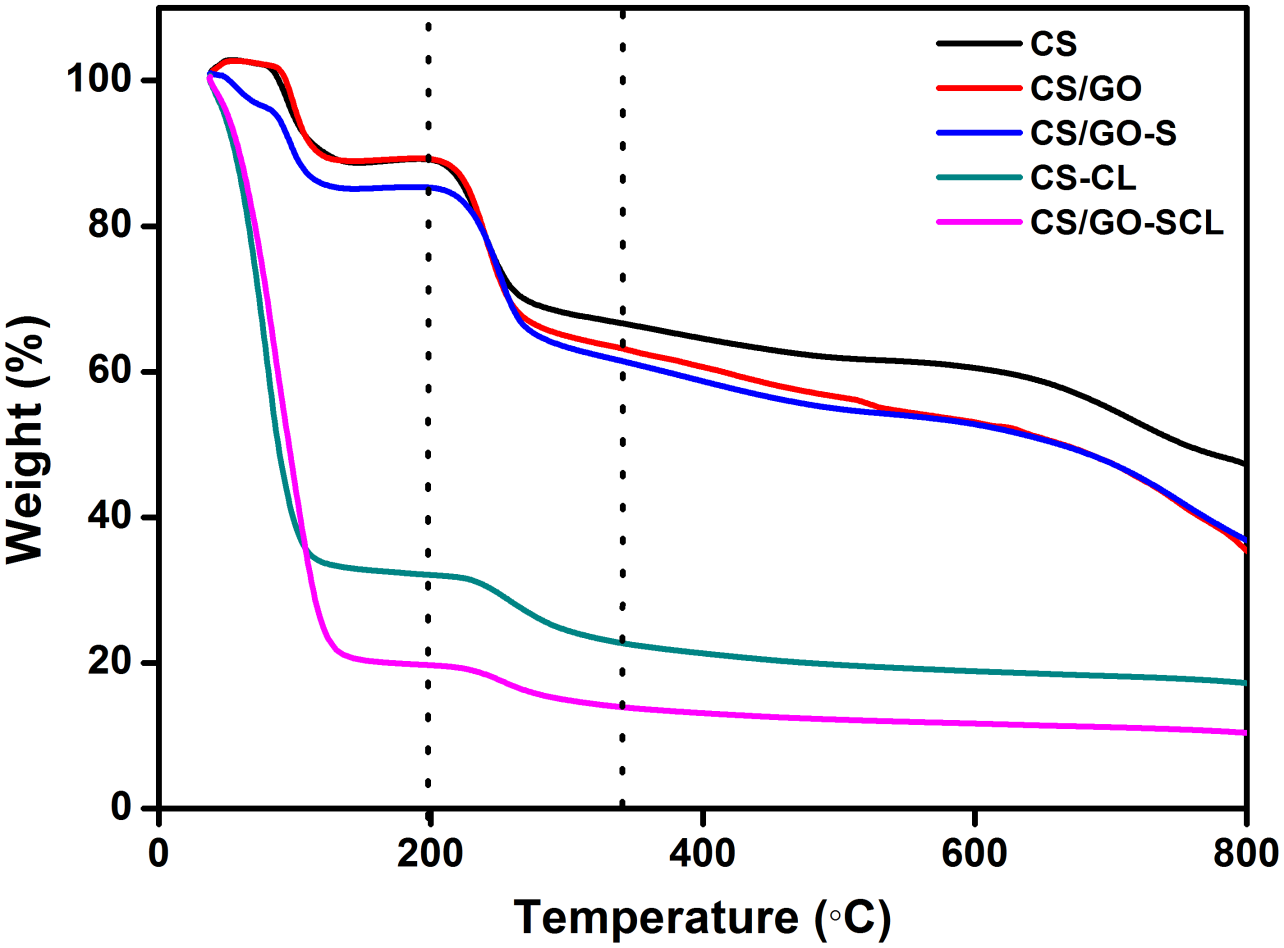
Thermogravimetric analysis results for prepared samples.

The thermal analysis of all the samples is clearly divided into 3 regions (**Fig. 5**). The first region from 40°C to 200°C [33], [34] indicates the loss of water molecules, whereas the second region from 200°C to 350°C [34], [35] is mainly because of the decomposition of the polysaccharide units of CS. The third region after 350°C indicates the degradation of 2surface –COOH groups [36], which was contributed by GO. For CS-CL and CS/GO-SCL, the water left behind with the matrix upon immobilization is reflected in the massive loss of water in the first region. The loss of lipase also contributed to this large decrease [33]. The weight difference trends for the second region were similar for all 5 curves regardless of the lipase immobilization, which implied that lipase was immobilized through physisorption [33].

**Figure 6:**
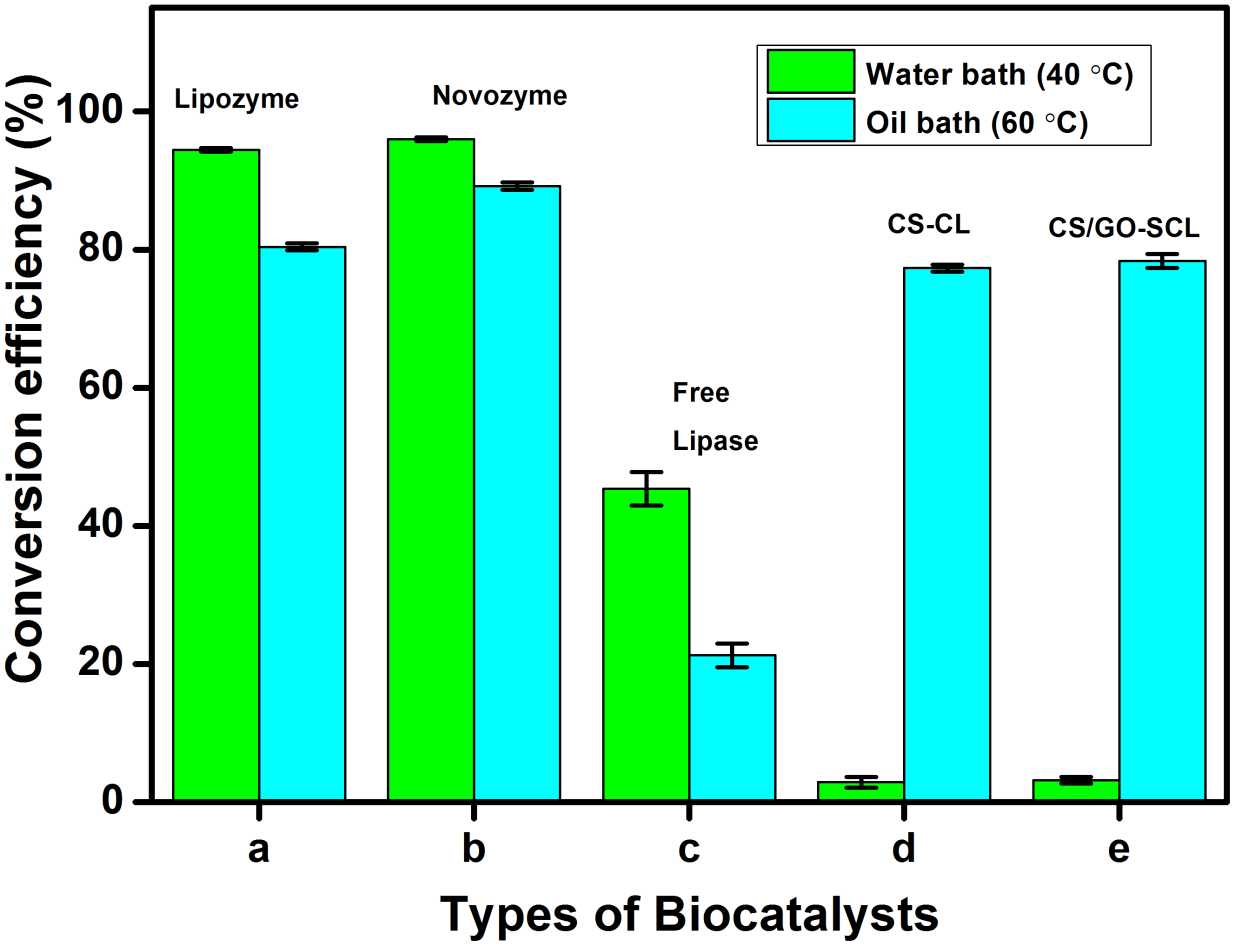
Lipase activities for lipozyme, novozyme, free lipase, CS-L, CS/GO-SL, CS-CL, and CS/GO-SCL.

Based on **Fig. 6**, the CS-L and CS/GO-SL possessed nearly the same lipase activities, which were 3.23 and 3.48 unit, respectively. This shows that the involvement of GO caused little increase in the lipase activity but improved the mechanical strength of the support [23]. During the lipase immobilization steps, the CS/GO-SL beads were able to maintain their shapes after 24 h of shaking, but some CS-L beads had their structures ruptured. The dismally low lipase activity was tremendously enhanced after using the coupling agents because the use of NHS and EDC can enhanced the lipase immobilization onto supports [37]. The CS-CL beads possessed a higher absorbance, with a calculated activity of 107.40 unit, compared to CS/GO-SCL beads, which had a lower enzyme activity of 63.53 unit. The significantly high activity of the coupled CS-CL and CS/GO-SCL could be explained in two ways. Initially, the biomimetic surface layer of the coupled beads was able to create a biocompatible environment for the immobilized lipase and thus retain the high lipase activity. Then, the hydrophilic biomimetic layer could expose the active sites of the lipase and reduce the random coupling of lipase and the beads [37]. The importance of hydrophilicity is clearly portrayed by the minimal enzyme activity of the commercial lipozyme and novozyme. During the enzyme assay, the hydrophobic lipozyme and novozyme adhered to the walls of the vials, which caused a lipase loss and data inaccuracy. The free lipase possessed a lipase activity of 15.22 unit. This proved that the beads had successfully increased the lipase activities by exposing the active sites upon immobilization onto the polymer matrix and thus acted as a good biocatalyst support [37].

**Figure 7:**
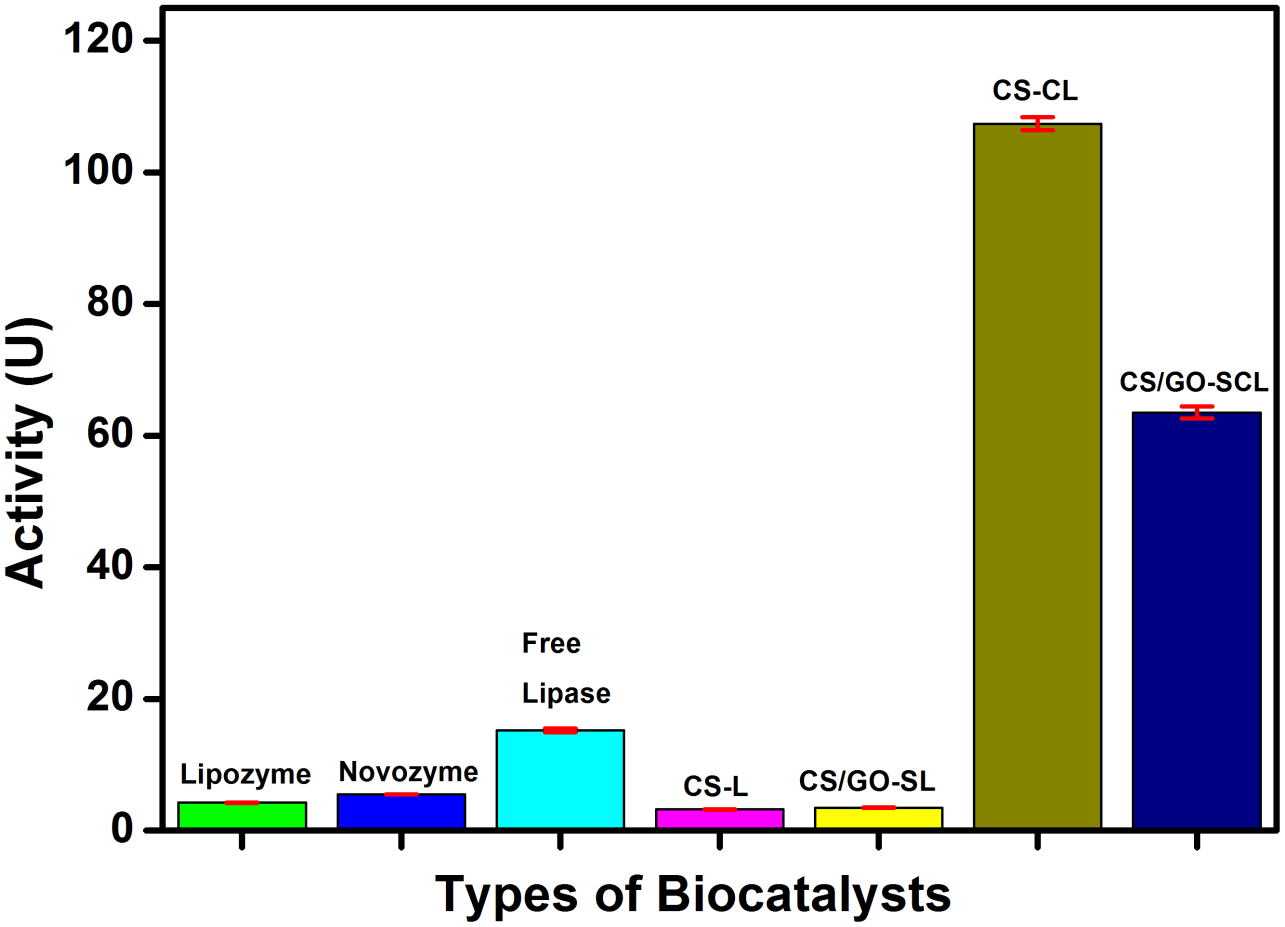
Percentage of conversion into wax ester after esterification process between lauric acid and oleyl alcohol catalyzed by (a) lipozyme, (b) novozyme, (c) free lipase, (d) CS-CL, and (e) CS/GO-SCL using water bath (on right) and oil bath (on left) methods.

**Fig. 7** shows the percentage of esterification catalyzed at two different temperatures of 40°C and 60°C, as controlled by water bath [27] and oil bath [28] methods. The novozyme and lipozyme showed the highest conversions of ∼96% and ∼95% using the water bath method [38], [39]. The conversion efficiencies of the novozyme and lipozyme were significantly higher than those of the other biocatalysts. Free lipase had a 45% conversion, whereas CS/GO-SCL and CS-CL possessed 3.16% and 2.88%, respectively. The poor conversion of the beads could plausibly be due to the poor mixing among the reactants, solvent, and biocatalyst because the biocatalysts floated on the reaction mixture in the simple agitation state. In contrast, the commercial biocatalysts mixed thoroughly with the reactants and solvent because they were fully dispersed in the reaction medium. The immersion of the biocatalyst in the reaction medium was influenced by the density of the biocatalyst. The commercial biocatalysts consisted of compact beads with a diameter of 450 µm, whereas the prepared porous biocatalysts had an average diameter of 430 µm.

The conversion efficiencies of CS-CL and CS/GO-SCL using the oil bath method were 77% and 78%, respectively, because of the improved lipase activity in the biocatalysts. However, the physical integrity of CS-CL was compromised at 60°C because the beads crumbled significantly at the end of the reaction compared to that of CS/GO-SCL. In addition to the agitation state during the reaction, the heat energy at the higher temperature of 60°C influenced the rate of reaction by increasing the collision frequency between the enzyme and support [28]. Moreover, a higher temperature reduced the mixture viscosity and improved the support diffusion process. However, a further increase in the incubation temperature, to more than 65°C, would lead to enzyme denaturation [28]. Even though the lipozyme and novozyme maintained the highest conversion efficiencies of 80% and 89%, respectively, the water bath method was better than the oil bath method for the commercial biocatalysts. The commercial biocatalysts decomposed to nothing at the oil bath temperature of 60°C. This resulted in the release of lipase, which then recoiled to the state of neat lipase, causing the active sites of the enzyme to be hidden once again.

## Conclusion

Chitosan beads incorporated with sonicated GO provided a much improved platform for the immobilization of lipase compared to the non-sonicated GO. This was because the smaller size GO was well-dispersed within the matrix of the chitosan polymer, creating uniformity in the active sites throughout the beads. In the presence of coupling agents, NHS and EDC, the lipase activity improved as much as 5 fold and 3 fold for chitosan and CS/GO, respectively. The CS beads containing GO had a much better mechanical strength compared to those of the neat CS and commercially available lipozyme and novozyme because the CS/GO beads almost maintained their initial physical appearance after the vigorous esterification between lauric acid and oleyl alcohol. Even though the thermal analysis showed that lipase was physically adsorbed onto the prepared beads, they possessed the high catalytic activity of esterification. The conversion efficiency of the prepared CS/GO biocatalyst was more than 85% with the commercially available lipozyme and novozyme. Considering that the cost of 10 g of lipozyme is 400 USD, whereas that of novozyme exceeds 900 USD, the prepared lipase immobilized beads could provide a cheaper alternative for lipase usage.
