# Correction: Enhanced Biocatalytic Esterification with Lipase-Immobilized Chitosan/Graphene Oxide Beads

**DOI:** 10.1371/journal.pone.0114093

**Published:** 2014-11-14

**Authors:** The PLOS ONE Staff

The eighth author’s name is spelled incorrectly. The correct name is: Chin Hua Chia.
